# Removing Metal Ions from Water with Graphene–Bovine Serum Albumin Hybrid Membrane

**DOI:** 10.3390/nano9020276

**Published:** 2019-02-16

**Authors:** Xiaoqing Yu, Shuwei Sun, Lin Zhou, Zhicong Miao, Xiaoyuan Zhang, Zhiqiang Su, Gang Wei

**Affiliations:** 1 State Key Laboratory of Chemical Resource Engineering, Beijing Key Laboratory of Advanced Functional Polymer Composites, Beijing University of Chemical Technology, Beijing 100029, China; yu_xiaoq@163.com (X.Y.); 2017200438@mail.buct.edu.cn (S.S.); 18810867810@163.com (Z.M.); 2 School of Chemical Engineering and Technology, Tianjin University, Tianjin 300072, China; linzhou@tju.edu.cn; 3 Otto Schott Institute of Materials Research, Friedrich Schiller University Jena, Löbdergraben 32, 07743 Jena, Germany; 4 Faculty of Production Engineering, University of Bremen, D-28359 Bremen, Germany

**Keywords:** protein, self-assembly, graphene oxide, membrane, water purification

## Abstract

Here we report the fabrication of graphene oxide (GO)-based membranes covalently combined with bovine serum albumin (BSA) for metal ions detection. In this system, BSA acts as a transporter protein in the membrane and endows the membrane with selective recognition of Co^2+^, Cu^2+^, AuCl_4_^−^, and Fe^2+^. Combining the metal-binding ability of BSA and the large surface area of GO, the hybrid membrane can be used as a water purification strategy to selectively absorb a large amount of AuCl_4_^−^ from HAuCl_4_ solution. Moreover, BSA could reduce the membrane-immobilized AuCl_4_^−^ by adding sodium borohydride (NaBH_4_). Interestingly, adsorption experiments on three kinds of metal ions showed that the GO–BSA membrane had good selective adsorption of Co^2+^ compared with Cu^2+^ and Fe^2+^. The morphology and composition changes of the membrane were observed with atomic force microscopy (AFM) and Raman spectroscopy, respectively. It is expected that this facile strategy for fabricating large-scale graphene-biomolecule membranes will spark inspirations in the development of functional nanomaterials and wastewater purification.

## 1. Introduction

Purification of wastewater is always the focus of industry, pharmaceutical enterprises, and environmental protection departments [1]. Wastewater contains not only dyes but also metal ions, such as Co^2+^, Cu^2+^, and Fe^2+^ [2]. In addition, the wastewater from gold purification contains residual AuCl_4_^−^, which causes severe healthcare problems for human beings [3]. Therefore, the separation of precious metal ions from wastewater is of great significance for wastewater purification, and to some extent, for metal recovery. Among various biomolecules, proteins have been suggested as a potential candidate for removing heavy metal ions from water [4,5,6]. For instance, Mezzenga et al. [7,8] reported that β-lactoglobulin amyloid fibril could be combined with activated carbon and used for the efficient removal of heavy metal ion from water. Their findings indicated that these protein fibrils allowed the reduction of membrane-immobilized metal ions into valuable metal nanoparticles. Another work showed that bovines serum albumin (BSA) can reduce Au(III) ions into Au clusters in the presence of NaOH [9]. 

As a typical single atomic two-dimensional nanomaterial, graphene exhibits a large specific surface area and high charge mobility, which endows it with applications in material science [10,11], nanotechnology [12], analytical science [13,14], biomedicine [15,16,17], wastewater purification [18,19,20], and other fields [21]. In particular, chemically-modified graphene nanomaterials, such as graphene oxide (GO) [22,23,24], reduced graphene oxide (RGO) [25,26,27], and graphene quantum dots (GQDs) [28,29], can improve water dispersion [30,31,32]. Graphene oxide and RGO were reported to be capable of effective water restoration and adsorption of toxic gases [33]. On the one hand, the oxygen-containing functional groups on the surface of GO, such as carboxyl and hydroxyl groups, can adsorb metal cations to achieve water purification. On the other hand, the products left can be easily separated from water [34]. In order to selectively adsorb the pollutants in water [35], many GO hybrid nanomaterials have been prepared with molecules such as polymers [36], peptides [37], proteins [38], and other small molecules [39]. Superior to other molecules, proteins become excellent biomolecules to bind with GO because of their low cost and rich functional groups. Most recently, Lu et al. [10] applied β-lactoglobulin on the surface of RGO sheets. The composite was used as a template for gold nanoparticle (AuNP) assembly. These AuNPs assembled on the β-lactoglobulin–RGO composites yielded an improved surface-enhanced Raman scattering (SERS) for Rhodamine 6G (Rd6G). In other cases, BSA can also be combined with GO to efficiently detect and extract Hg^2+^ [40]. Therefore, fabricating GO-protein composite membranes is of great value for reducing metal ions to metal nanoparticles and further achieving the biomimetic membrane in sewage treatment [35]. 

**Figure 1:**
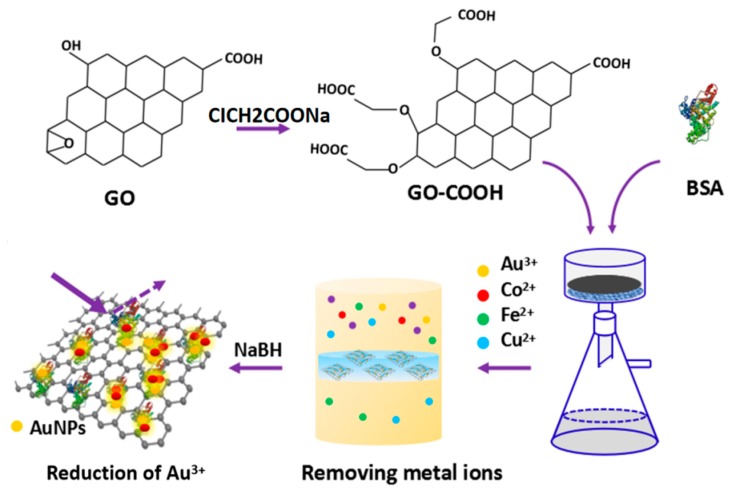
Schematic illustration of the fabrication of graphene oxide (GO)–bovines serum albumin (BSA) hybrid membrane for absorbing metal ions and reduction of metal ions.

Inspired by the above studies, we provide a facile method to fabricate the GO–BSA membrane for removing heavy metal ions from water. Firstly, the GO prepared by the Hammers method was mixed with ClCH_2_COONa and NaOH to transform the esters, hydroxyl groups, and epoxies into carboxyl groups on the surface of GO. Then we fabricated the GO–BSA membrane by incorporating BSA on GO–COOH via vacuum filter (Figure 1). The GO–BSA membrane was used to remove metal ions, i.e., AuCl_4_^−^, Co^2+^, Fe^2+^, and Cu^2+^ ions from water. 

## 2. Materials and Methods 

### 2.1. Chemicals and Materials

Natural graphite flake (99.8% purity) was purchased from Sigma–Aldrich. Chloroauric acid (HAuCl_4_·3H_2_O, ≥49.0% Au basis), disodium hydrogen phosphate (Na_2_HPO_4_), sodium dihydrogen phosphate (NaH_2_PO_4_), phosphoric acid (H_3_PO_4_), sulfuric acid (H_2_SO_4_), HCl, NaBH_4_, potassium permanganate (KMnO_4_), BSA, ClCH_2_COONa, N-ethyl-N’-(3-dimethylaminopropyl) carbodiimide hydrochloride (EDC), and N-hydroxysuccinimide (NHS) were purchased from J&K company (Beijing, China). Ethanol was purchased from Beijing Chemicals Co., Ltd. (Beijing, China). All the chemicals used are at least of analytical grade and directly used without additional purification. The water purified through a Millipore system (18.2 MΩ·cm) was used throughout. 

### 2.2. Synthesis of GO–BSA Nanohybrids

Graphene oxide was prepared from natural graphite flake by a modified Hummers method as described in our previous reports [22]. At first, in order to convert the ester group, hydroxyl group and epoxy group on the surface of GO to carboxyl group, GO was blended with ClCH_2_COONa and HCl. Briefly, 2 g NaOH and 2 g ClCH_2_COONa were added to 50 mL GO solution of 2 mg/mL concentration simultaneously. After sonication for 2 h, the product was neutralized by 1M HCl, followed by repeated centrifugation and rinsing to obtain GO–COOH products. The precipitation was again dispersed to 100 mL PBS (phosphate buffer saline) buffer (pH = 6.1). The second step is to make BSA react with GO–COOH, mainly by using –NH_2_ of BSA and –COOH of GO–COOH. The specific operation was as follows, 50 mL GO–COOH (1 mg/mL) was dissolved in the PBS buffer solution. Then 20 mM EDC and 5 mM NHS were added and kept stirring for 1 h at room temperature. After that, 100 mg BSA in 10 mL PBS buffer solution (pH = 6.1) was added in the solution. Finally, the product was centrifuged and rinsed for three times, and then dialyzed for three days. The water was replaced three times a day to remove the inorganic ions. All the steps were conducted under room temperature to remove the unreacted BSA, EDC, NHS, and the ions in the buffer solution.

### 2.3. Fabrication of GO–BSA Membranes 

The GO and GO–BSA membranes were fabricated by the well-introduced vacuum filtration method previously described [7,8]. In brief, GO and GO–BSA solutions were diluted to 1 mg/mL before filtrating. The as-prepared solutions were filtered through a 0.22 μm polyethersulfone (PES) membrane and then dried under 60 °C. The GO membrane and GO–BSA membrane were obtained with a load of about 5 mg/cm^2^.

### 2.4. Removing Metallic Ions with Membranes

The absorbance of the solution before and after the membrane filtration was measured by using an ultraviolet spectrophotometer (PerkinElmer, America). 5 mM HAuCl_4_, 0.1 M CoCl_2_, NiCl_2_, and CuCl_2_ were configured, respectively. Metal ionic solutions were extracted with a 5-mL syringe containing the GO–BSA membranes. The filtrate was collected with a 2-mL centrifuge tube. The absorbance of the solution before and after filtration was measured several times. The GO–BSA membranes should maintain the same concentration and volume during the experiment to reduce experimental errors. 

### 2.5. Characterization Techniques

A scanning electron microscope (SEM, JSM-6700F, JEOL) and a transmission electron microscope (TEM) were used to characterize the surface morphology changes before and after GO and BSA hybridization. The preparation procedures of the SEM samples were: dry GO powder, attach it to conductive adhesive, and then tape the GO–BSA hybrid membrane onto the conductive adhesive. The TEM images were acquired on a JEM-2100F field transmission electron microscopy (Hitachi Limited, Japan) at an acceleration voltage of 200 kV. To prepare the TEM samples, the GO and GO–BSA were dispersed in deionized water by ultrasound for 1 h, and then a 5 μL solution was dripped on the copper grid with natural drying. The cpper grids for TEM characterization were purchased from the Plano GmbH (Wetzlar, Germany). Atom force microscopy (AFM) images were acquired on Bruker MultiMode 8 (Bruker, Beijing, China) in tapping mode. The type of AFM probe was a standard silicon tips (RTESP, Bruker, Beijing, China) with a spring constant of 40 N/m. The silicon wafer used for AFM was cleaned and dried with acetone and deionized water. The samples were diluted to a certain concentration and dripped on the silicon wafer with naturally drying. A Raman spectrometer (HORIBA Scientific, France) was used to characterize the sample using a laser source with a center wavelength of 780 nm. In the Raman spectroscopy measurement, the solid sample was placed directly on the quartz glass. The absorbance of the solution before and after the membrane filtration was measured using an ultraviolet spectrophotometer (PerkinElmer, America). X-ray diffraction (XRD) was taken on a Rigaku D/max-2500 VB+/PC equipment (BRUKER AXS GMBH, Germany) with a scanning 2θ angle of 5° to 90° at a voltage of 40 kV and a current of 40 MA. 

## 3. Results and Discussion

### 3.1. Morphological Characterization of GO–BSA Membranes

**Figure 2:**
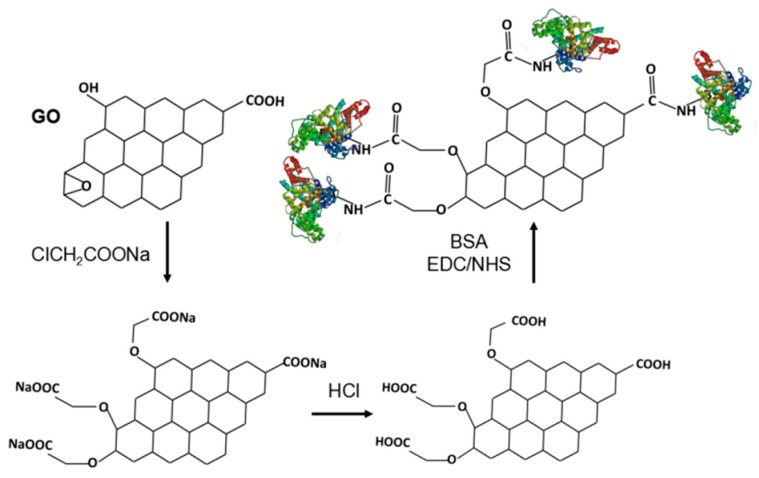
Schematic illustration of the functionalization of GO and the formation of GO–BSA nanohybrids by covalent interaction-mediated biomolecular self-assembly on GO surface.

To increase the efficiency of the conjugation between GO and BSA, we increased the content of the carboxyl group on the GO surface according to a previous work [35]. The oxygen-containing functional groups such as epoxy, ester, and hydroxyl on the surface of GO were effectively converted to –COOH by adding ClCH_2_COONa under alkaline conditions, as shown in Figure 2. It was reported that the –NH_2_ contained in His, Arg, and Lys groups as well as the N-terminal of BSA can bind to –COOH of GO–COOH in the presence of EDC/NHS [41].

**Figure 3:**
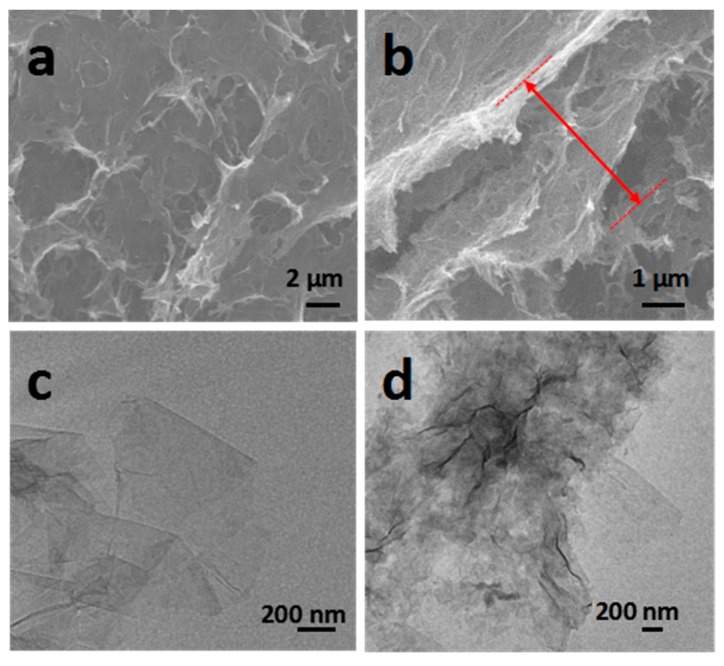
Morphological analysis of GO–BSA membranes: (**a**) SEM image of surface morphology of the GO–BSA film. (**b**) SEM image of the cross-section of the GO–BSA. (**c**) TEM image of surface morphology of the GO. (**d**) TEM image of surface morphology of the GO–BSA.

The morphology and thickness of the fabricated GO–BSA membranes were first characterized by SEM, and the images are shown in Figure 3a,b. It can be found that the surface of the thin membranes presented some irregular holes, indicating that the fabricated membranes were not packed tightly. The cross-section of the GO–BSA membranes indicated the typical wrinkled lamellar structure. The thickness of the membranes was measured to be around 3 ± 1 μm. Due to the addition of protein molecules into the interlayer of GO, the loose structure inside the GO–BSA could be seen from the section diagram.

For comparison, the GO membrane was prepared with the same technique. The TEM image in Figure 3c showed that the GO, prepared by the modified Hummers method, displayed a thin lamellar structure with an area of hundreds of nanometers. When combined with BSA, the atoms on the surface caused a change of light and shade contrast. It can be clearly seen in Figure 3d that the surface of GO was transformed from the original smooth state into a rough surface structure. It indicates that a thin layer of protein was adsorbed onto the GO surface, which confirmed the successful functionalization of GO with BSA.

### 3.2. Structural and Property Characterizations of GO–BSA Membranes

**Figure 4:**
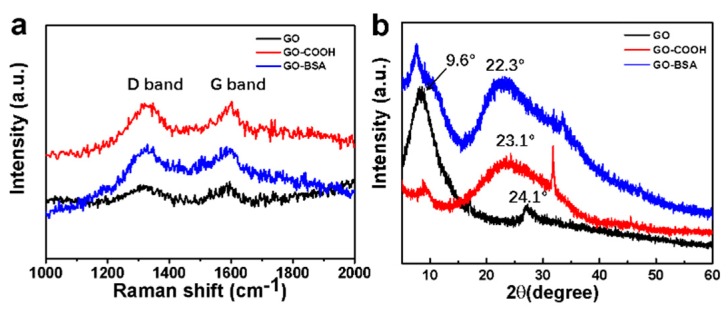
Structural and property characterizations of GO–BSA membranes: (**a**) Raman spectra of GO and GO–BSA hybrid membrane. (**b**) XRD patterns of the GO and GO–BSA hybrid membrane.

The Raman spectrum was further used to characterize the change of the functional groups before and after the GO hybridization with BSA, as shown in Figure 4a. The Raman spectrums were measured by using a laser source with the center wavelength of 780 nm. Two peaks at 1315 cm^−1^ and 1585 cm^−1^ can be assigned to the D and G bands, respectively. The D band is related to the vibrations of sp^3^ carbon atoms of disordered GO nanosheets, and the G band corresponded to the vibrations of sp^2^ carbon atom domains of graphite. The GO–COOH Raman spectra exhibited that there was enhanced D peak at 1380 cm^−1^ (Figure 4a) and G-peak at 1576 cm^−1^ (Figure 4a), as well as no 2D peak at 2700 cm^−1^ (not shown) in the Raman spectrum, indicating that all GO nanosheets were functionalized as GO–COOH [42]. Furthermore, the intensity ratio between D and G bands (I_D_/I_G_) of GO was 0.95 and further increased to 1.01 for GO–COOH. This trend indicated the presence of disordered structures and defect sites on GO–COOH sheets, most likely resulting from the carboxylation of GO [43]. After the combination with BSA, the peak of the D at 1315 cm^−1^ and the G at 1585 cm^−1^ decreased compared with GO–COOH. The I_D_/I_G_ increased from 1.01 to 1.11, indicating the defect density increases as a result of exfoliation and chemical modification [44,45]. Therefore, we suggested that BSA could not only be inserted into the GO nanosheets but can also interconnect GO monolayers together to form condensed GO–BSA hybrid membranes.

The atomic and molecular structures of GO, GO–COOH, and GO–BSA hybrid membranes were determined by XRD, shown in Figure 4b. The characteristic diffraction peak at 9.6° represents the (001) planes of GO [46]. A diffraction peak located at approximately 9.6° in XRD pattern of GO–COOH was also observed, corresponding to the crystal plane (001) of exfoliated GO [43]. After the formation of GO–BSA composite, the (001) planes still existed but seemed to be weaker compared with GO, illustrating the change of oxygen-containing groups. The broad peak of (002) planes located at 24.1° indicated the disordered stacking structure of the GO layers. The same peak located at 22.3° in the GO–BSA composites demonstrated that the GO–BSA composite had much more disordered stacking structures than GO and GO–COOH.

### 3.3. Separation of Metal Ions by GO–BSA Membranes

The BSA contained more than ten types of amino acids and was rich in lots of functional groups, such as imidazolyl and thiol groups (–SH), making it easy to adsorb heavy metal ions [40]. It had been reported that there was an interaction between BSA and gold ions involving biomineralization or biomimetic mineralization [9,37]. Since BSA contained 21 Tyr residues and possibly other residues with reduction functionality, it can reduce Au^3+^ through their phenolic groups, and their reducing power can be improved by adjusting the pH. For example, AuCl_4_^−^ can be reduced to Au(0) by biomolecules under alkaline conditions or in the presence of NaBH_4_. Therefore, the prepared GO–BSA membranes are assumed to adsorb AuCl_4_^−^ in water and can reduce it to AuNPs. 

**Figure 5:**
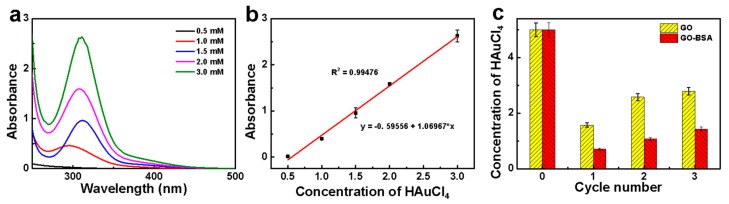
Removing AuCl_4_^−^ ions: (**a**) UV-Vis spectra of the HAuCl_4_ with different concentrations, (**b**) the relationship between UV absorbance and HAuCl_4_ concentration at 310 nm, and (**c**) calculation of the concentration of HAuCl_4_ after the filtration according to the standard curve. Inset: the illustration of the color change before and after the filtration of the GO–BSA membrane.

As shown in Figure 5a, the UV profiles were performed based on different concentrations of HAuCl_4_ solution. It can be seen that HAuCl_4_ exhibited obvious absorption peaks at 310 nm, mainly due to the electron transition of Cl atoms to Au. The relationship between the absorbance and the concentration was fitted in Figure 5b. The linear correlation coefficient was about 0.99476, showing a good linear relationship. After the first and second rounds of the HAuCl_4_ filtration (Figure 5c), there was almost no absorption intensity at 310 nm. This indicated that the GO–BSA membrane can effectively adsorb AuCl_4_^−^. But a weak absorption peak appears at 310 nm after the third cycle. Due to the partial detachment of AuCl_4_^−^ from the membrane and the increased concentration of AuCl_4_^−^, leading to the occurrence of characteristic peaks. According to the standard curve in Figure 5b, it can be calculated that the concentration of AuCl_4_^−^ after each filtration was 0.71 mM of the first filtration, 1.07 mM of the second filtration, and 1.43 mM of the third filtration, respectively. Particularly, for the first filtration, the efficiency was measured to up to 85 ± 1%. For the third filtration, part of the AuCl_4_^−^ fell off from the membrane, resulting in the increase of AuCl_4_^−^ concentration in the filtrate (light yellow in color), as shown in Figure 5d. Although GO had a certain adsorption capacity for AuCl_4_^−^, it can be seen from the Figure 5d that the GO–BSA had a better adsorption effect than it, which indicated that BSA had a better adsorption capacity for AuCl_4_^−^. In addition, although the stability of the membrane was relatively well under normal conditions, the performance of the GO–BSA membrane will be affected at high or low temperature due to the biological molecules it contained. Moreover, the GO–BSA membrane was also vulnerable to large impact.

**Figure 6:**
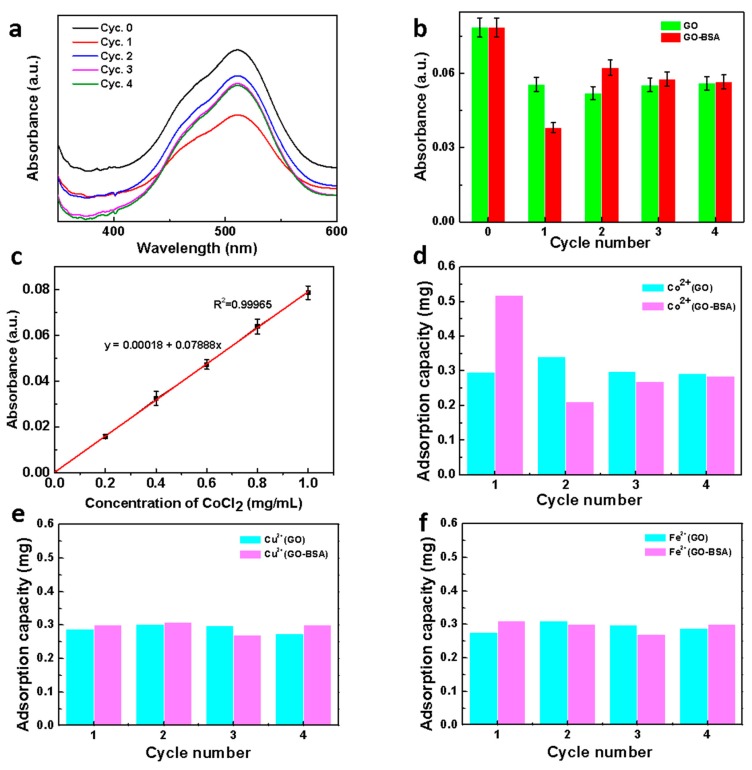
Removing other metallic ions: (**a**) the UV spectra of CoCl_2_ before and after adsorbing by GO–BSA membrane; (**b**) the absorbance of CoCl_2_ at 511 nm before and after adsorbing by GO–BSA membrane; (**c**) the standard curve of CoCl_2_ at 511 nm; (**d**) the adsorption capacity of GO and GO–BSA filter membrane to CoCl_2_; (**e**) the adsorption capacity of GO and GO–BSA filter membrane to CuCl_2_. (**f**) The adsorption capacity of GO and GO–BSA membrane to FeCl_2_.

The separation performances of several metal cations were determined via a similar method. The UV absorption of CoCl_2_ appears at 511 nm (Figure 6a), which mainly resulted from the charge transfer between Co^2+^ and Cl^−^. For the first filtration, the absorbance intensity was significantly decreased, demonstrating the CoCl_2_ was adsorbed by the GO–BSA membrane. For the second filtration, Co^2+^ started to desorb from the membrane due to some weak binding between Co^2+^ and GO-BSA (Figure 6b). For the further filtration round, the absorbance was almost unchanged, indicating that the adsorption of Co^2+^ on the membrane achieved saturated. Based on the relationship between the absorbance and concentration of CoCl_2_, a standard curve can be drawn (Figure 6c) with good linear fitting. The adsorption capacity of each filtration can be calculated according to the linear equation. Compared with the GO membrane, the adsorption capacity of GO–BSA at the first filtration was greater (Figure 6d), indicating that the functional groups on the protein had a certain effect on the binding of Co^2+^. 

In order to demonstrate the selective adsorption of the GO–BSA membrane towards Co^2+^, we explored the adsorption capacity of the GO–BSA membranes to other metal ions, including Cu^2+^ and Fe^2+^. Figure 6e,f present the adsorption capacities of the GO and GO–BSA membranes to Cu^2+^ and Fe^2+^, respectively. This obtained results indicated that the GO–BSA membranes had no significant selective adsorption to Cu^2+^ and Fe^2+^. In this process, BSA, as a metal binding protein, played the role of ion transporter. According to previous reports [35], Co^2+^ easily forms a stable combination with carboxylate and nitrogen, such as aspartate (Asp, D). While Cu^2+^ leaned toward nitrogen and sulfur ligands, such as histidine (His, H), cysteine (Cys, C), and methionine (Met, M) amino acid ligands. In addition, the similar adsorption amount of Cu^2+^ and Fe^2+^ may be related to ligand coordination geometry. The Co^2+^ ions showed a preference for octahedral while Cu^2+^ ions were easier to bind square-planar coordination geometry. The certain functional groups on GO–BSA, such as imidazole, can combine with Co^2+^ but not Cu^2+^ and Fe^2+^, leading to selective adsorption of Co^2+^. After several cycles, the adsorbed metal ions may fall off and enter into the filtrate, which led to the decrease of the adsorption capacity after the first cycle.

The metal ions adsorption performances of GO–BSA membranes were significantly enhanced attributing to the abundant functional groups on BSA molecules. Recent work by Zhang et al. [47] fabricated red-blood-cells like BSA/Zn_3_(PO_4_)_2_ hybrid particles via the one-step method based on coordination between BSA and zinc ion. The adsorption efficiency of the hybrid particles increased with time from 86.33% (5 min) to 98.9% (30 min). The adsorption capacity was 6.85 mg/g at optimal conditions. They demonstrated that the hybrid particles displayed excellent adsorption properties on Cu^2+^ due to the high amount of BSA and Zn ions coordinated in the particles. This result proved the great potential of BSA-based nanomaterials applied in heavy metal ion treatment. A similar trend was presented in our system: GO–BSA membranes. Interestingly, the maximum adsorption capacity for Cu^2+^, 0.3 mg/mg, was much larger than the hybrid particles in previous work. The higher adsorption capacity can be attributed to the combination of the large-surfaced GO and abundant functional groups of BSA molecules. 

### 3.4. Reduction of Au^3+^ on GO–BSA Membranes 

**Figure 7:**
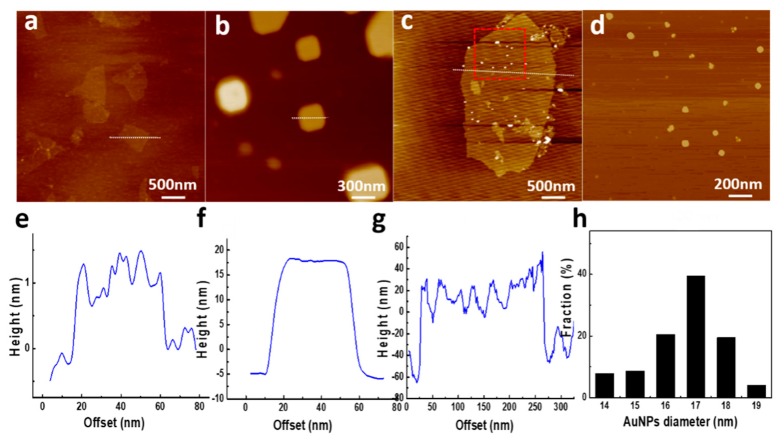
The AFM height images and corresponding section analysis of (**a**) GO, (**b**) BSA immobilized on GO, and (**c**) GO–BSA–Au, as well as their extracted line profiles of (**a**) GO, (**b**) BSA immobilized on GO, and (**c**) GO–BSA–Au, respectively. (**d**) AuNPs immobilized on the GO–BSA membrane. (**e**) Height image of extracted line profiles in (**a**) GO. (**f**) Height image of extracted line profiles in (**b**) BSA immobilized on GO. (**g**) Magnified height image of the red square area in (**c**), showing the AuNPs immobilized on the GO–BSA membrane. (**h**) The size distribution of AuNPs.

The hybrid membrane was also assumed to reduce the adsorbed metallic ions to metal NPs. As a high-resolution imaging technique, AFM uses the relationship between the probe and the sample to detect the surface topography of the sample and obtain the size of AuNPs. Compared to pure GO with a thickness of around 1 nm (Figure 7a,e), the GO–BSA nanosheets exhibit a larger thickness within 20 nm. The significant increase of thickness can be attributed to the immobilization of BSA molecules on the GO–COOH nanosheets. Bovine serum albumin, as a serum albumin protein, displays globular conformation with a diameter of 14 nm under physiological conditions [48]. The increase of the nanosheet thickness after treatment with BSA indicates that lots of BSA molecules are immobilized on the surface and edges of GO–COOH (Figure 7b,f). 

As was shown by AFM results in Figure 7b,c, the nanosheet thicknesses of the BSA immobilized GO–COOH and the GO–BSA–Au significantly increased. Meanwhile, the homogenous layer of BSA and BSA–Au can be observed on the surfaces of GO–COOH. This indicates that the saturation time of two hours is sufficient for GO–COOH fully-covered with BSA. Due to the limited COOH group on GO surfaces, the BSA immobilized on the GO–COOH surfaces contains not only covalently-bonded BSA molecules but also irreversibly adsorbed BSA molecules.

After treatment with NaBH_4_, the thickness of GO–BSA–Au was measured to be around 30 nm. This indicates a large number of AuNPs grow on the surface of the GO–BSA nanosheets. The formation of AuNPs suggests that the adsorbed AuCl_4_^−^ was reduced to Au (Figure 7c). The selected profile of the corresponding AFM height image provides the dimension information of the GO sheets and AuNPs, as shown in Figure 7g. The size distribution of the AuNPs was relatively uniform (Figure 7h). Thus, the AuNPs that anchored on the surface of GO–BSA nanosheets have an average size of 17 ± 2 nm. 

Liu et al. [49] adsorbed BSA onto GO/RGO and deposited Au nanoparticles on BSA using HCIO_4_. In comparison to their work, rather than assembling AuNPs directly onto the membrane, GO–BSA was a facile method without the involvement of latex (i.e., polystyrene sphere) assemblies. Besides, biological molecules in the GO–BSA membrane were not easy to desorb during the reduction of Au^3+^. AuNPs can be stabilized by a combination of Au–S bonding with the protein (via the 35 Cys residues in BSA), and the steric protection due to the bulkiness of the protein [35]. The function of BSA in this process can also be confirmed by the work of Xie et al. [9], who reported that BSA can reduce AuCl_4_^−^ to Au (0) in an alkaline environment. The difference was that their reduced products were subnanometer-sized Au clusters, while most of the products we reduced were AuNPs. Besides, it can maintain a good adsorption capacity after repeated cycles. This indicated that the stability of the GO–BSA membrane was relatively well.

## 4. Conclusions

In summary, we reported that GO–BSA hybrid membranes can be used to remove metal ions from water. Due to the high metal-binding capacity of BSA and the large surface area of GO, the membrane can selectively absorb AuCl_4_^−^ from solutions. In addition, the reduction of AuCl_4_^−^ to AuNPs was successfully achieved after adding NaBH_4_. This membrane was also demonstrated to selectively adsorb Co^2+^, but not Cu^2+^ and Fe^2+^. As a sewage treatment membrane, it was of great significance for removing heavy metal ions from water.
